# Children at risk: A nation-wide, cross-sectional study examining post-traumatic stress symptoms in refugee minors from Syria, Iraq and Afghanistan resettled in Sweden between 2014 and 2018

**DOI:** 10.1186/s13031-020-00311-y

**Published:** 2020-10-02

**Authors:** Øivind Solberg, Alexander Nissen, Marjan Vaez, Prue Cauley, Anna-Karin Eriksson, Fredrik Saboonchi

**Affiliations:** 1 Division for Implementation and Treatment Research, Norwegian Centre for Violence and Traumatic Stress Studies, P.box 181, 0409 Oslo, Nydalen Norway; 2 Department of Health Science, Swedish Red Cross University College, Stockholm, Sweden; 3 Division for Forced migration and Refugee Health, Norwegian Centre for Violence and Traumatic Stress Studies, Oslo, Norway; 4 Division of Insurance Medicine, Department of Clinical Neuroscience, Karolinska Institutet, Stockholm, Sweden; 5 Unit of Mental Health, Children and Youth, The Public Health Agency of Sweden, Solna, Sweden

**Keywords:** Refugee minors, Posttraumatic stress disorder, Afghanistan, Syria, Iraq

## Abstract

**Background:**

The objective of the present study was to assess nation-wide, representative prevalence estimates for symptom-defined posttraumatic stress disorder (PTSD) within populations of refugee minors from Afghanistan, Syria and Iraq resettled in Sweden.

**Methods:**

A nation-wide, cross-sectional, questionnaire study with a stratified sample of refugee minors, aged 16–18 years, from Afghanistan, Iraq and Syria, resettled in Sweden between 2014 and 2018 (*N* = 5071) was conducted. The response rate was 22.3%, leaving *n* = 1129 refugee minors (boys 53.1% / girls 46.9%) in the final sample. Symptom-defined prevalences of PTSD were measured using CRIES-8 with ≥17 as cut-off. Data were analyzed using frequency distributions, and strata-specific PTSD prevalences with 95% confidence intervals (95% CIs), were estimated. The association between migratory status on arrival (unaccompanied vs. accompanied) and PTSD was estimated using crude and adjusted odds ratios (OR) utilizing logistic regression analyses with 95% CIs.

**Results:**

Overall, the weighted PTSD prevalence was 42% (95% CI 38.9–45.1), with minors from Afghanistan presenting the highest prevalence (56.9, 95% CI 51.5–62.2), compared to minors from Iraq (36.8, 95% CI 28.9–45.4) and Syria (33.4, 95% CI 29.4–37.6). Unaccompanied minors from Afghanistan had higher odds of PTSD compared to accompanied minors from Afghanistan (OR = 1.92, 95% CI 1.08–3.40). Gender differences were non-significant.

**Conclusions:**

High prevalences of symptom-defined PTSD among refugee minors in general and in unaccompanied minors from Afghanistan in particular, were revealed. Findings calls for continued efforts to support this especially vulnerable group.

## Background

According to the United Nations Children’s Fund (UNICEF), 170,000 unaccompanied refugee minors— youth under 18 years of age separated from their parents/guardian—applied for asylum in Europe in 2015 and 2016 [1]. Often withheld at border-crossings, beaten, forced to sleep outdoors, and left without access to basic needs, these refugee children, throughout their flight, are especially at risk [2].

Indeed, research focusing on refugee children and unaccompanied refugee minors has consistently reported heightened risk for mental health disorders, including posttraumatic stress disorder (PTSD), within this population and existing evidence suggests that mental health disorders tend to be highly prevalent, even several years after resettlement [3–12]. Prior research has shown that a number of factors are associated with the development of mental health problems in refugee children and adolescents, such as exposure to violence, stress in the post-migration context, family stress, separation from parents, and discrimination [13–16]. The host country environment has also been shown to be particularly important in the determination of longer-term mental health outcomes and adaptation to the country of resettlement [16]. However, although scientific knowledge about this vulnerable population has increased over the years, recent reviews have highlighted the need for further examination, due to high variability within reported findings and methodological inconsistencies in the design and measures used [17, 18]. This includes a wide range of prevalence levels for PTSD (range: 19–60%), and other mental health disorders and long-term outcomes [19, 20]. In fact, there is a notable lack of consensus among researchers regarding the long-term outcomes of mental health problems in this population, and clear findings regarding estimates of long-term prevalences are currently lacking [19, 20]. However, a few studies have suggested that, while the mental health of refugee youth previously diagnosed with depression or PTSD may improve slightly over time, metal health problems are likely to still be present at follow up [19]. It has also been suggested that PTSD and depression can lead to poor performance in school, which may in turn lead to a higher likelihood of engagement in risky behaviour [19]. Studies focusing on mental health in minors have also largely been based on small, non-random samples, often with predominantly unaccompanied, male participants [8, 21–26], highlighting an underlying problem of convenience sampling. Such samples are often non-representative and might, in some cases, provide biased or incomplete information about the populations in question [17]. According to Kien and colleagues (2018), the research field therefore currently lacks reliable prevalence data for several European countries, including Sweden. In sum, the current lack of representative and methodologically consistent studies suggests that research conducted with large, randomly selected samples, and standardized, validated measures, in order to improve consistency and generalizability of findings, is highly warranted [17, 20, 27].

Findings related to gender differences among unaccompanied refugee minors have also thus far been largely inconclusive. Although female gender has been shown to be associated with a heightened vulnerability towards certain mental health problems, a lack of representative studies and relevant data for male refugees should encourage caution when interpreting findings [28, 29]. Still, refugee and asylum-seeking females are typically disproportionately vulnerable to threats such as sexual exploitation and abuse, as well as associated negative mental health outcomes [29, 30]. This seems to hold true for girls as well as women, with studies finding that underage girls experienced exposure to sexual violence at a far greater rate than boys, and that they were found to show more symptoms of anxiety and behavioral problems than their male counterparts [31]. This vulnerability may be a significant factor that contributes to the overrepresentation of males in samples of unaccompanied refugee minors - families may simply feel that it is safer to send their boys than their girls on the dangerous journey to Europe [9].

Previous studies also differ considerably, both in where they are conducted and in the nationalities that are studied [17]. The asylum-process, legal frameworks and integration policies used in each country are known to vary greatly and the different refugee populations are by no means equivalent in terms of pre-flight and migratory experiences. Furthermore, the proportions of accompanied and unaccompanied minors within the refugee populations differ widely and differences in education levels, socio-economic status, religious and sociocultural values all contribute to variations in post-migratory stress levels and affect mental health outcomes [32]. Considering this, the field is in need of representative studies including different refugee populations from the same region, resettled in one country, under one legal framework, with both accompanied and unaccompanied minors, in order to establish methodologically more sound comparative analyses.

In 2015, European countries received unusually high numbers of asylum-seekers from Syria, Afghanistan, and Iraq. According to Eurostat [33], the highest number of unaccompanied minor applicants was registered in Sweden (approx. 35,000 unaccompanied minors, 40% of the EU total). In the EU, entry procedures for unaccompanied refugee minors who lodge an application for asylum are well-established and more-or-less harmonized across member states. In Sweden, an asylum application is submitted to the Swedish Migration Board and, in most cases, entry is permitted for unaccompanied refugee minors. A key component of the reception arrangements for unaccompanied refugee minors is the appointment of a guardian, or a custodian, and/or public counsel. The appointed guardian acts not only to provide legal support in the asylum process, but also with regard to other aspects of the unaccompanied refugee minors stay. Minors seeking asylum in Sweden have the right to attend school on the same terms as other children living in the country and to study at upper secondary school level as long as these studies begin before they turn 18 years old. Asylum-seeking minors also have the right to medical care and dental care on the same terms as other children living in Sweden (Swedish Migration Agency, Children in the asylum process). Accommodation and other care facilities for unaccompanied refugee minors is provided, with the type of accommodation varying depending on the minors’ individual needs and age.

Considering the aforementioned, the objective of the present study was to assess nation-wide, representative prevalence estimates for symptom-defined PTSD within populations of refugee minors from Afghanistan, Syria and Iraq resettled in Sweden. We hypothesized that PTSD prevalences for unaccompanied minors would be significantly higher compared to accompanied minors, and that the PTSD prevalence for unaccompanied girls would be significantly higher, compared to boys. Considering that one out of two unaccompanied minors originates from Afghanistan, PTSD prevalence estimates were expected to vary by country, with minors from Afghanistan reporting the highest PTSD prevalences, due to the expected large proportion of unaccompanied minors within this subsample and the high symptom burden linked to being unaccompanied. In order to overcome some of the above-mentioned methodological shortcomings, we used a well-known, validated and methodologically comparable measure for symptom-defined PTSD within a large, randomly drawn sample of resettled refugee minors in Sweden.

## Method

### Design, sampling and participants

**Fig. 1:**
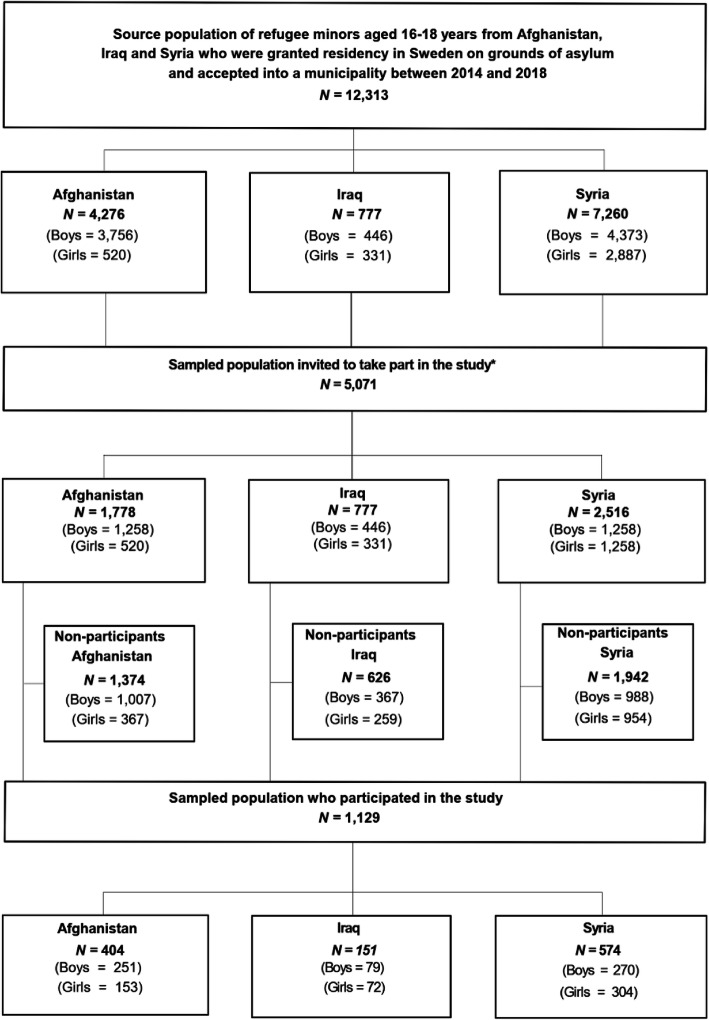
Country-specific number of refugee minors in source population, number sampled and number participating. * In strata with less than 1258 individuals, all individuals were sampled. For strata with 1258 or more individuals, simple random sampling was used to select a total 1258 individuals

The present study has a cross sectional design. The source population of the study consisted of a known and completed population of *N* = 12,313 girls and boys aged 16 to 18 years from Syria, Iraq and Afghanistan who were granted residency in Sweden on grounds of asylum, and were accepted into a municipality between 2014 and 2018 (See flow chart, Fig. 1). The source population was identified through the Total Population Register (TPR), covering every individual that has resided in Sweden on a permanent basis held by Statistics Sweden. The source population was stratified by gender and country of birth resulting in a total of 6 strata. From this stratified source population, a sample of *n* = 5071 refugee minors (41% of the complete source population), was drawn and included in the study (see power analysis). The following sampling strategy was used. From the three smallest strata (girls from Afghanistan, and both boys and girls from Iraq) containing less than *n* = 845 (*n* = 5071 divided by 6 strata), all refugee minors were included. For the remaining three largest strata (boys from Afghanistan, and both boys and girls from Syria, *n* = 3774), simple random sampling was used to select *n* = 1258 from each stratum. The rationale for including all refugee minors in the three smallest strata, i.e. to oversample these strata, was to increase the precision of stratified estimates, and to guard against the risk of small sample sizes from these strata of the source population, which may have occurred if proportional allocation for sampling had been applied [34].

#### Power analysis

This study is an age-specific sub-study of a larger, nation-wide, cross-sectional questionnaire study conducted in Sweden in 2018 (Refugee Children and Adolescence’s Health, RCAH), the source population of which consisted of all refugee minors (aged 12 to 18 years) from Syria, Iraq and Afghanistan who were granted residency in Sweden on grounds of asylum and accepted into a municipality between 2014 and 2018 (*N* = 25,584). The initial power analysis for this study was done with the overall aim of estimating the prevalence of common mental health problems in the target population with adequate precision. Due to large heterogeneity of previously reported estimates of common mental health problems among refugee minors [35], the power analyses were replicated with two hypothesized point prevalence estimates of 20% and 50%. Based on an estimate of 20%, the analysis indicated that a sample size of *n* = 1537 would be needed for 0.02 precision at a 95% confidence level. The analysis also indicated that this sample size of approximately 1500 individuals would provide sufficient power for estimation of a higher prevalence at 50%, albeit with slightly less precision (d = 0.025). Considering the expected non-response rates inherent in survey studies of refugee populations, this would require a 30% response rate of n ≈ 5000 (15% for *n* = 10,000) from the sample. As these response rates were deemed achievable, and with the consideration of the logistical constrains of the study, the sample size of *n* = 10,000 for RCAH study, with approximately *n* = 5000 between 16 to 18 years old, which corresponds to a substantive proportion (41%) of the total source population, was selected.

An information letter explaining the purpose of the study and what participation entailed was sent out to the sampled population on June 5th 2018 and the actual questionnaire was distributed via postal mail on June 12th, 2018, with a pre-paid return envelope enclosed. Two reminders were sent out to non-responders: the first on June 26th (reminder card only); and the second on August 31st (reminder card and questionnaire). Information about the study was also presented online using Swedish Red Cross University College’s webpages and featured in an online magazine targeting refugee adolescents and Swedish youth in general. Addresses were re-checked and updated prior to posting the second reminder. Enclosed with the questionnaire was an informed consent form where it was explicitly stated that participation was voluntary and that submission of the questionnaire implied consent. The information provided also included a phone number participants could call if they had any questions or concerns related to the study.

The study was approved by the Stockholm Regional Ethical Review Board (*Approval number: 2018/456–31/5*). Statistics Sweden was responsible for conducting the survey. Data was analyzed anonymously.

### Adaptation and development of the questionnaire

A standard double-blind translation and back-translation procedure was used for linguistic adaptation of the questionnaire to Arabic and Dari/Farsi for all sections except where pre-validated and adapted scales already existed. Cognitive testing by *verbal probing* was performed; i.e. an interview in which a series of probe questions in regard to respondents’ potential difficulties in comprehension, retrieval of information and judgment and responses to the questionnaires’ content, is administrated by an interviewer. This was performed for each linguistic version of the questionnaire, with *n* = 15 native Arabic and Dari/Farsi speaking children. In cases of detecting difficulties, the content of the questionnaire was discussed with professional interpreters as well as native speaking community members. Following these procedures, minor adjustments in wording and phrasing of some of the content of the questionnaire were performed.

#### Symptom-defined posttraumatic stress disorder (PTSD)

Symptom-defined posttraumatic stress disorder (PTSD) was assessed through the Child Revised Impact of Event Scale (CRIES-8) [36]. The scale is frequently used to measure PTSD among unaccompanied refugee minors [17, 18, 30] The scale consists of eight items measuring intrusion (four items) and avoidance (four items) in the last 7 days related to a stressful life event. Each item is scored on a 4-point frequency scale (0 = Not at all; 1 = Rarely; 3 = Sometimes; 5 = Often), giving a total score ranging from 0 to 40. In the present study, a total score ≥ 17 defined a PTSD case, i.e. minors with a mean scored equal to or above 17 were considered to have PTSD like symptoms or as being at risk for PTSD, regardless of how many items were answered. A cut-off score of 17 is in accordance with previous studies on unaccompanied refugee minors [22]. In order to minimize the risk of inflating PTSD prevalence estimates, we decided to include all individuals who had answered at least one item on the scale. By doing so, we included a higher number of non-PTSD participants in the denominator of the prevalence estimate fraction, hence avoiding inflation. Participants with all 8 items missing were excluded (6,4%).

#### Migratory status on arrival

The variable used to define a participant as an unaccompanied vs. accompanied refugee minor was provided by Statistics Sweden’s database STATIV (www.scb.se). The STATIV database is developed by Statistics Sweden together with the Swedish Integration Board to provide data within different areas of society from an integration policy perspective. Specifically, STATIV states that *“an unaccompanied refugee minor is defined as a refugee under 18 years of age who arrives in Sweden without a parent or other legal guardian”.*

#### Living situation

A potentially important factor for unaccompanied refugee minors is whether they are reunited with their mother and/or father or other family member in the host country. Presumably, the potential negative impact of fleeing as an unaccompanied minor may be partly remedied if one is reunited with family/relatives, or, conversely, exacerbated if one is not. We therefore decided to include living situation in the present study and asked all participants which adults they lived with. There were 8 possible answer choices: 1. *I live with both my mother and father*; 2. *My parents do not live together and I live with my mother most/all of the time*; 3. *My parents do not live together and I live with my father most/all of the time*; 4. *My parents do not live together and I live equally with my mother and father*; 5. *I live with one or more adult relatives*; 6. *I live with an unrelated adult/adults*; 7. *I live in a family home or equivalent*; 8. *I live in an HVB-home*. A HVB-home is government funded institution/home where unaccompanied minors temporarily live and receive basic support. Answer categories 1–5 were combined into the category “*With mother and/or father or relatives”* due to few observations in some categories. Likewise, categories 6 and 7 were combined into the category “*Family home or unrelated adults”.*

#### Demographics

Data on gender and age at the time of assessment were obtained through the Statistics Sweden population registry.

### Statistical analyses

The data set was first cleaned and checked for errors and missing values. The number of missing values for a given variable can be gauged from the tables. Frequency distributions were calculated through cross-tabulations. Point estimates with 95% confidence intervals (95% CIs) of symptom-defined PTSD prevalences were calculated across strata of covariates (i.e. gender, age, migratory status on arrival and living situation), both for the total sample and the sample split by refugees’ country of origin. Point estimates were not calculated if there were less than 5 individuals in a cell when cross-tabulating covariates with PTSD. In order to correct for potentially bias in PTSD prevalence estimates caused by study design and non-response, weighted prevalence estimates (with 95% CI) was also calculated for the total sample and the sample split on country of birth using post-stratification weights [37]. In post-stratification weighting, strata-specific prevalences in the sample are weighted to reflect the source population’s distribution across strata. The strata used for post-stratification weights in the present study were based on the study design with the stratified source population (i.e. six strata in total). Chi-square tests for categorical variables were used to compare differences only in symptom-defined PTSD prevalence across country of birth, age gender, migratory status, and living situation.

Logistic regression analysis was used in order to estimate the association between migratory status on arrival (unaccompanied vs. accompanied) and symptom-defined PTSD, and is presented as crude and adjusted odds ratios (ORs) with 95% CIs and *p*-values from Wald test of equal odds. Regression models were built in a two-step, forward approach starting with crude associations and then adding age and gender as potential confounders, both for a priori reasons. Only individuals without missing values for all model variables were included in analyses, and the total number contributing data to a given model is noted in Table 3. Due to limited numbers of unaccompanied refugee minors from Iraq and Syria, and the fairly similar gender and age distribution in these subsamples, the two countries were combined in regression analyses in order to increase power.

No pre-registered document, with detailed plans for analyses, exists. Specific strategies for analyses were determined with data at hand. We have therefore attempted to keep analyses simple and transparent in order to reduce the chance of pursuing random noise in the data which could result from including large numbers of candidate variables and multiple testing [38]. Still, the broader study objectives and hypotheses were prospectively outlined in written documents, such as ethics application and study protocol.

## Results

Of the 5071 sampled refugee minors, 1129 returned the questionnaire for an overall response rate of 22.5% (See Fig. 1 for details). Country-specific response rates were: Afghanistan 22.7% (404 of 1778); Iraq 19.4% (151 of 777); and Syria 22.8% (574 of 2516). Descriptive statistics for the participants are presented in Table 1. There was a clear difference between refugee minors from Afghanistan compared to Syria and Iraq, with participants from Afghanistan both older and with a higher proportion of boys. Most notably, however, was that the refugee minors from Afghanistan were much more likely to be unaccompanied (over 60% compared to around 10% for Syria and Iraq), and live in a family home, with unrelated adults or in an HVB home (i.e. not with their mother and/or father or other relatives).

**Table 1:** Characteristics of study sample of resettled refugee minors in Sweden, by total and country of birth^1^

	Total sample ***N*** = 1129		Syria ***N =*** 574		Iraq ***N =*** 151		Afghanistan ***N =*** 404	
	***n***	(%)	***n***	(%)	***n***	(%)	***n***	(%)
**Gender**								
Boys	600	(53.1)	270	(47.0)	79	(52.3)	251	(62.1)
Girls	529	(46.9)	304	(53.0)	72	(47.7)	153	(37.9)
Total	1129	(100.0)	574	(100.0)	151	(100.0)	404	(100.0)
**Age**								
16	347	(30.7)	202	(35.2)	54	(35.8)	91	(22.5)
17	376	(33.3)	207	(36.1)	42	(27.8)	127	(31.4)
18	406	(36.0)	165	(28.7)	55	(36.4)	186	(46.1)
Total	1129	(100.0)	574	(100.0)	151	(100.0)	404	(100.0)
**Migratory status on arrival**								
Accompanied	805	(71.3)	511	(89.0)	139	(92.1)	155	(38.4)
Unaccompanied	324	(28.7)	63	(11.0)	12	(7.9)	249	(61.6)
Total	1129	(100.0)	574	(100.0)	151	(100.0)	404	(100.0)
**Living situation**								
With mother and/or father or relatives	832	(78.7)	545	(97.5)	135	(94.4)	152	(42.8)
Family home or with unrelated adults	112	(10.6)	10	(1.8)	6	(4.2)	96	(27.0)
HVB home^2^	113	(10.7)	4	(0.7)	2	(1.4)	107	(30.2)
Total	1057	(100.00)	559	(100.0)	143	(100.0)	355	(100.0)

^1^ Newly resettled refugee minors defined as refugees aged 16 to 18 years who were granted residency in Sweden on grounds of asylum and accepted into a municipality between 2014 and 2018

^2^ HVB-home is a government funded institution/home where unaccompanied minors temporarily live and receive basic support

Regarding the differences in distribution between respondents and non-respondents, there were no significant differences in regard to country of birth (*p* = 141). Boys were somewhat less likely than girls to participate (59.9% boys among non-respondents vs. 53.1% boys among respondents, *p* < 0.01), and there were slight differences in regard to age (16 years: 28% among non-respondents vs. 30.7% among respondents, 17 years: 31.9% among non-respondents vs. 33.3% among respondents, 18 years: 40.1% among non-respondents vs. 36.0% among respondents, *p* < 0.05). Unaccompanied refugee minors comprised 28.7% of the respondents while the corresponding proportion among non-respondents was 38.2% (*p* < 0.001). Assessment of Cohen’s h effect size, however, revealed that all differences in proportions between respondents and non-respondents were small to negligible (h = 0.030–0.084 for age, h = 0.137 for gender, h = 0.202 for unaccompanied migratory status).

The estimated prevalence of symptom-defined PTSD for total sample was 42.0% in the weighted analysis (95% CIs: 38.9–45.1; see Table 2). Country-specific analysis showed that refugee minors from Afghanistan had a higher prevalence of symptom-defined PTSD (56.1%) compared to refugee minors from Syria (33.6%) and Iraq (37.1%) (chi-square *p*-value < 0.001). Weighted country-specific prevalence estimates differed only marginally from unweighted estimates. There was no significant difference in symptom-defined PTSD prevalence between Syria and Iraq (chi-square *p*-value = 0.44). Furthermore, there was no evidence of a difference between symptom-defined PTSD prevalence between boys and girls, neither in the total sample (chi-square *p*-value = 0.36), nor within any of the countries (*p*-values = 0.73, 0.30 and 0.41 for Syria, Iraq and Afghanistan, respectively). In the sample as a whole, the results showed that unaccompanied refugee minors had higher symptom-defined PTSD prevalence than accompanied ones (53.7% vs. 37.1%; chi-square *p*-value < 0.001), although the prevalence in the unaccompanied group was markedly smaller in weighted analysis (43.1%). When comparing PTSD prevalences in unaccompanied vs. accompanied refugee minors within countries, there was only statistical evidence that the two groups differed in participants from Afghanistan (chi-square *p*-value = 0.035). There was no evidence of a difference in PTSD prevalence between unaccompanied and accompanied refugee minors within the subsample from Syria (chi-square p-value = 0.74). No statistical test was done for the subsample from Iraq because of insufficient number of observations.

**Table 2:** Prevalences of symptom-defined posttraumatic stress disorder (PTSD) and 95% confidence intervals (95% CI), by total and country of birth among participating resettled refugee minors in Sweden

	Symptom-defined PTSD prevalence							
	Total sample^**2**^ (***n =*** 1057)		Syria (***n =*** 551)		Iraq (***n =*** 132)		Afghanistan (***n =*** 374)	
	%	(95% CI)	%	(95% CI)	%	(95% CI)	%	(95% CI)
**Unweighted**	42.0	(39.1–45.0)	33.6	(29.7–37.6)	37.1	(29.3–45.7)	56.1	(51.1–61.1)
**Weighted**^**3**^	42.0	(38.9–45.1)	33.4	(29.4–37.6)	36.8	(28.9–45.4)	56.9	(51.5–62.2)
**Gender**								
Boys	43.3	(39.3–47.4)	32.8	(27.4–38.8)	32.8	(22.5–45.1)	57.7	(51.4–63.9)
Girls	40.5	(36.2–44.9)	34.3	(29.0–39.9)	41.5	(30.1–54.0)	53.3	(44.8–61.6)
**Age**								
16	38.7	(33.4–44.3)	30.1	(23.9–37.1)	42.2	(28.5–57.2)	57.0	(45.8–67.5)
17	42.5	(37.5–47.7)	33.0	(26.9–39.8)	39.5	(25.1–55.9)	59.7	(50.6–68.1)
18	44.2	(39.3–49.2)	38.3	(31.1–46.0)	30.6	(19.2–45.0)	53.4	(46.0–60.7)
Total								
**Migratory status on arrival**								
Accompanied	37.1	(33.7–40.6)	33.8	(29.7–38.1)	37.2	(29.0–46.2)	48.9	(40.4–57.4)
Unaccompanied	53.7	(48.1–59.1)	31.7	(21.4–44.3)	^a^		60.2	(53.8–66.2)
**Living situation**								
With mother and/or father or relatives	37.2	(33.9–40.7)	^a^		^a^		53.0	(44.5–61.3)
Family home or with unrelated adults	56.4	(46.9–65.4)	^a^		^a^		60.6	(50.4–70.0)
HVB home	56.9	(47.4–65.9)	^a^		^a^		57.3	(47.5–66.5)

^1^ A PTSD case was defined as a total score ≥ 17 on the CRIES-8 questionnaire

^2^ There were 72 individuals with missing for the PTSD variable: 23 from Syria; 19 from Iraq; and 30 for Afghanistan

^3^ Prevalence for total sample was weighted using direct standardization – i.e. strata-specific sample prevalences were weighted according to the distribution of the source population across strata. There were six strata in total, defined by all possible combinations of gender and country of origin

^a^ Not calculated because there were less than 5 individuals in individual cells when PTSD was stratified by living situation

When exploring the association between migratory status on arrival (unaccompanied vs. accompanied) and symptom-defined PTSD with logistic regression, there was some evidence that unaccompanied refugee minors from Afghanistan had higher odds of PTSD compared to accompanied minors from Afghanistan after adjusting for age and gender (OR = 1.92; 95% CI 1.08–3.40; *p-*value 0.026). Age and gender were both negative confounders of this relationship with the crude OR biased towards the null (see Table 3). There was no evidence of an association between migratory status and PTSD in either unadjusted or adjusted models for Syria and Iraq (combined in analyses for power issues). Similarly, there was no evidence that gender was associated with symptom-defined PTSD.

**Table 3:** Logistic regression analyses with crude and adjusted odds ratios (ORs) with 95% confidence intervals (95% CI) for the association between migratory status on arrival (unaccompanied vs. accompanied) and symptom-defined posttraumatic stress disorder (PTSD)

	Syria and Iraq (***n =*** 683)			Afghanistan (***n =*** 374)		
	OR	(95% CI)	***p***-value^**1**^	OR	(95% CI)	***p***-value^**1**^
**Unadjusted model:**						
**Migratory status:** unaccompanied	0.91	(0.55–1.53)	0.73	1.58	(1.03–2.42)	0.036
**Adjusted model**						
**Migratory status:** unaccompanied	0.93	(0.55–1.56)	0.78	1.92	(1.08–3.40)	0.026
**Gender:** girl	1.11	(0.81–1.53)	0.52	1.25	(0.71–2.20)	0.44
**Age** (vs 16): 17	1.07	(0.73–1.57)	0.74	1.06	(0.59–1.91)	0.84
18	1.18	(0.80–1.75)	0.41	0.77	(0.44–1.33)	0.34

^1^ *p*-value from Wald test of equal odds

As living situation and migratory status on arrival were strongly correlated (*r =* 0.75), there was a risk of multicollinearity issues if both variables were added to the same logistic regression model. With this risk highlighted, when living situation was included in the adjusted regression model for Afghanistan, the OR for PTSD comparing unaccompanied to accompanied refugee minors changed from 1.92 to 2.14, though confidence intervals were wide (95% CI 0.87–5.27) and results not significant at the 0.05 level (*p-*value for Wald test of equal odds = 0.1, not shown in table).

## Discussion

The objective of the present study was to assess nation-wide, representative prevalence estimates for symptom-defined PTSD within populations of refugee minors from Afghanistan, Syria and Iraq resettled in Sweden. We hypothesized that symptom-defined PTSD prevalences for unaccompanied minors would be significantly higher compared to accompanied minors, and PTSD prevalences were expected to vary by country, with minors from Afghanistan reporting the highest prevalences. We also hypothesized that PTSD prevalences among unaccompanied girls would be significantly higher, compared to boys. Overall, our findings revealed a notably high number of “at risk” minors with prevalences rates of PTSD above 40%, with refugee minors from Afghanistan presenting the highest estimates, indicating the presence of PTSD in over half (56%) of these children.

With approximately 4 out of 10 refugee minors presenting symptoms indicating PTSD, a far greater, multifold burden was reflected in the present study, compared to available epidemiological evidence pertaining to prevalence of PTSD in high-income countries [39, 40]. This also holds true when comparing our estimates to reported prevalence estimates for trauma exposed children and adolescents [41]. Still, in a recent study conducted with refugee minors in a German context, a greater proportion of participants than in the current study were deemed to present post-traumatic stress symptoms above the determined cut-off (56.1% in comparison with 42% in the present study) [42]. Notably, these findings are equivalent to those for the subset of the present study’s population of minors from Afghanistan (56% prevalence for symptom-defined PTSD), a population highly represented in the German study cited. These results are also comparable with a similar study which also used a sample of refugee minors primarily from Afghanistan, resettled in Norway (58.7% prevalence of symptom defined PTSD) [43].

Still, exposure to war- and conflict-related trauma may present a particularly complex case. Indeed, the prevalence estimates previously reported for children in proximity of war have been shown to be markedly high [44], close to the rates detected in the present study. In fact, war- and conflict-related experiences may be particularly detrimental to children’s mental health due to the concurrent erosion of supportive resources and networks, such as those provided by parents and family [45, 46]. Following this line of thought, the estimated high prevalences of PTSD in the present study may reflect the pronounced likelihood of exposure to war and conflict and the concurrent detrimental effects that follow, leaving unaccompanied minors at particular risk.

In this regard, the significantly higher prevalence of PTSD presented by minors from Afghanistan, compared to those from Syria and Iraq, stands out. Since the situations in Afghanistan, Syria and Iraq are characterized by severe and long lasting conflicts and war, and given that the eastern Mediterranean migration route has been the most important pathway for refugees from the Greater Middle East, including minors from Afghanistan, it is not obvious that we can ascribe the observed differences to mere specificities of the pre-migratory factors [47]. The main differences in the distribution of risk and protective factors in our study sample, however, seem to be related to the minors’ migratory status and current living situation. Refugee minors from Afghanistan were to a larger extent unaccompanied and most live without a parent or relative. Living without a parent or relative post arrival in host-country has previously been shown to significantly increase the risk for the development of psychopathology [48]. From our available data, thus, the post-migratory living conditions may provide an explanation for the heightened PTSD prevalence among this group of refugee minors. Furthermore, going beyond the legal definition of “unaccompanied”, which only denotes if a legal guardian was present at arrival or not, unaccompanied minors from Afghanistan currently living without a parent or relative, in this sense, might more fully represents respondents who actually traveled alone, i.e. unaccompanied in the non-legal meaning of the word, hence reporting the highest symptom burden. In other words, minors who traveled unaccompanied in the legal sense could still have been traveling with several members of their family or friends (but not legal guardians) and be reunited with their guardians in Sweden under the laws of family reunification. These children are classified as unaccompanied either way by the Swedish authorities. On the other hand, unaccompanied respondents currently living alone in family homes/HVB-homes would be more likely to truly have traveled alone and unprotected. Children put in HVB-homes, do not have a legal guardian or are still waiting to be reunited with their guardians. Most of them probably also traveled without close family and friends who could provide for them after arrival in Sweden. In this sense they were and are more alone/less protected both during their flight and after arrival in Sweden, compared to those living with family or friends, and thus expected to report higher symptom levels.

It is also worth emphasizing that participants in the present study were granted residency between the years of 2014 and 2018. This implies that these children may well have been living in Sweden for up to 5 years at the time of our data collection. The high rates of “at risk” children presented are therefore particularly concerning, as they raise questions regarding whether the PTSD prevalences have declined over time, remained stable or even increased post arrival. Prior longitudinal studies exploring the mental health of unaccompanied refugee minors have found sustained levels of PTSD symptoms over time, even while other measures of mental disorders declined [8, 49]. While the present study did not collect longitudinal data, the findings herein might be consistent with these cited studies.

Finally, somewhat surprisingly, our hypothesis stating that PTSD prevalences among unaccompanied girls would be significantly higher, compared to boys, was not confirmed. The present study did not find any evidence that symptom-defined PTSD prevalence varied by gender, neither in the sample overall nor within subsamples from Afghanistan, Syria or Iraq. ​ As mentioned in the introduction, findings related to gender differences among unaccompanied refugee minors have thus far been largely inconclusive and a lack of representative studies and relevant data for male refugees calls for caution when interpreting previous findings [28, 29]. A possible explanation for the lack of PTSD differences between genders may be attributed to the fact that all minors, regardless of gender, are at the heightened risk of being exposed to extreme adversities, trauma and human right violations during their flight to Europe. In other words, within the context of flight, war and conflict, these children’s main vulnerability might lie in the compounding of exposures to extreme adversities and their young age, not their gender. The gendered pattern of risk and vulnerability factors among refugee minors within this specific context, thus, warrants future investigation, taking pre-, peri-, and post-migratory exposures and conditions into account.

### Limitations

As less than a quarter of invited refugee minors decided to participate in the present study, there is an obvious possibility that selection bias may have influenced results, in particular the study’s prevalence estimates. Using weighted analyses may partly correct for selection bias issues, however, it will not correct for problems with representativeness *within* strata. For example, if non-participation in the study was positively associated with poorer mental health as some evidence suggests [50]**,** the study may underestimate PTSD prevalence even in weighted analyses. Selection bias may also have affected the association between migratory status and PTSD, although exposure-outcome associations are generally considered to be quite robust to nonresponse [51, 52].

Another limitation is the use of self-reported data and the short-form version of the Impact of Event Scale (CRIES-8). This short-form version was chosen in order to ease the response burden of the participating children. However, CRIES-8 is a brief, child-friendly measure designed to screen children/youth at risk for PTSD. The scale therefore does not incorporate the DSM-IV A-criterion, i.e. it does not anchor the questions asked to a specific potentially traumatic event. The referenced “event” is instead “flight in general” (before, during and after flight) and all the potentially traumatic events such forced migration might entail. CRIES-8 also lack items measuring hyper-arousal, the third cluster of PTSD symptoms, as defined in DSM-IV. Together, this calls for caution when interpreting the prevalences presented in this study. However, CRIES-8 is a tool frequently used to screen for posttraumatic stress symptoms in children and has been validated for use with refugee children [21, 22]. Findings on PTSD from the present study will therefore be readily comparable to other relevant studies on refugee minors in the literature.

Finally, as discussed above, the categorization of refugee minors as unaccompanied vs. accompanied in the present study was based on a pre-existing variable obtained from Statistics Sweden and the following definition: “... a refugee under 18 years of age who arrived in Sweden without a parent or other legal guardian.” In other words, being unaccompanied in the present study did not necessarily mean that a refugee minor travelled alone. She/he could have travelled with family other than mother and father, and/or with friends. It is also worth noting that even though 63 refugee minors from Syria were categorized as unaccompanied, only 14 stated *not* to live with their mother and/or father or relatives. This contrasts with refugee minors from Afghanistan, where 203 of 249 unaccompanied refugee minors stated *not* to live with their mother and/or father or relative (i.e. a much higher proportion). Considering this, future research should explore our findings further within a longitudinal, mixed methods design in order to identify factors that exacerbate or alleviate PTSD symptoms over time within these subsamples. We also would like to note that our study only focused on minors who already had received a residence permit in Sweden. By doing so, the present study overlooks the vulnerable and distressing period of the asylum-seeking process itself and longitudinal data, incorporating this period, could have added additional clarity to our results. Regrettably, a longitudinal study including non-registered asylum-seeking children at the time of data collection was not logistically possible. It also needs to be highlighted that the present study incorporates a limited number of possible confounders and/or predictors of PTSD. A more thorough investigation of the links between pre-, peri- and post-migratory stress exposure, mental health, quality of life, integration, social inclusion, school- and work-participation, incorporating registry data per country of origin, would have been preferable in order to broaden the scope.

## Conclusions

To our knowledge, this is one of the first studies to report weighted PTSD prevalence estimates by country within a nation-wide, large, random sample, encompassing both unaccompanied and accompanied refugee minors from Afghanistan, Syria and Iraq within a high-income, EU country. Thus, our findings can provide the field with more robust estimates for PTSD prevalences among refugee minors in Europe. Unaccompanied minors from Afghanistan presented the highest PTSD prevalence and, according to Eurostat, are still the largest group of unaccompanied refugee minors in the EU [53]. Together, these findings call for continued efforts to support this especially “at risk” group, changes in immigration policies and the utilization of readily available interventions that specifically targets mental ill health. Furthermore, the overall high symptom defined prevalences of PTSD among minors reported in the present study, paints a harrowing picture of a group of children that are truly at risk and in need of international protection and care.

## Data Availability

In line with the Conflict and Health regulations and guidelines on data sharing, as well as Swedish law and ethical approval, the data from this study cannot be shared on an individual data level. Sharing this data would compromise the confidentiality of participants due to the small number of observations in individual cells and the sociodemographic characteristics in the results. Therefore, the used data is viewed as back-traceable and identifiable. Further, in addition to participants being part of a vulnerable group who shared personal and sensitive data, they did not consent to data being shared. Individual data sharing would breach the terms of consent. Therefore, the datasets generated and analysed during the current study are not publicly available, but aggregated data are available from the corresponding author on upon reasonable request.

## Abbreviations

UNICEF: United Nations children’s fund
URMs: Unaccompanied refugee minors
EU: European union
PTSD: Posttraumatic stress disorder

## References

[1] A child is a child: Protecting children on the move from violence, abuse and exploitation [Internet]. UNICEF. 2017 [cited 2019 Sep 30]. Available from: https://www.unicef.org/publications/index_95956.html.

[2] Menjívar C, Perreira KM (2019). Undocumented and unaccompanied: children of migration in the European Union and the United States. J Ethn Migr Stud.

[3] Derluyn I, Broekaert E (2008). Unaccompanied refugee children and adolescents: the glaring contrast between a legal and a psychological perspective. Int J Law Psychiatry.

[4] Thommessen S, Laghi F, Cerrone C, Baiocco R, Todd BK (2013). Internalizing and externalizing symptoms among unaccompanied refugee and Italian adolescents. Child Youth Serv Rev.

[5] Vervliet M, Meyer Demott MA, Jakobsen M, Broekaert E, Heir T, Derluyn I (2014). The mental health of unaccompanied refugee minors on arrival in the host country. Scand J Psychol.

[6] Seglem KB, Oppedal B, Raeder S (2011). Predictors of depressive symptoms among resettled unaccompanied refugee minors. Scand J Psychol.

[7] Bronstein I, Montgomery P, Ott E (2013). Emotional and behavioural problems amongst Afghan unaccompanied asylum-seeking children: results from a large-scale cross-sectional study. Eur Child Adolesc Psychiatry.

[8] Jensen TK, Skar A-MS, Andersson ES, Birkeland MS (2019). Long-term mental health in unaccompanied refugee minors: pre- and post-flight predictors. Eur Child Adolesc Psychiatry.

[9] Huemer J, Karnik NS, Voelkl-Kernstock S, Granditsch E, Dervic K, Friedrich MH (2009). Mental health issues in unaccompanied refugee minors. Child Adolesc Psychiatry Ment Health.

[10] Yalın Sapmaz Ş, Uzel Tanrıverdi B, Öztürk M, Gözaçanlar Ö, Yörük Ülker G, Özkan Y (2017). Immigration-related mental health disorders in refugees 5-18 years old living in Turkey. Neuropsychiatr Dis Treat.

[11] Ehntholt KA, Trickey D, Harris Hendriks J, Chambers H, Scott M, Yule W (2018). Mental health of unaccompanied asylum-seeking adolescents previously held in British detention centres. Clin Child Psychol Psychiatry.

[12] Michelson D, Sclare I (2009). Psychological needs, service utilization and provision of care in a specialist mental health clinic for young refugees: a comparative study. Clin Child Psychol Psychiatry.

[13] Fazel M, Reed RV, Panter-Brick C, Stein A (2012). Mental health of displaced and refugee children resettled in high-income countries: risk and protective factors. Lancet..

[14] Hjern A, Angel B (2000). Organized violence and mental health of refugee children in exile: a six-year follow-up. Acta Paediatr.

[15] Montgomery E. Long-term effects of organized violence on young Middle Eastern refugees' mental health. Soc Sci Med. 2008;67(10):1596–603. 10.1016/j.socscimed.2008.07.020. 10.1016/j.socscimed.2008.07.020 18755530

[16] Montgomery E (2010). Trauma and resilience in young refugees: a 9-year follow-up study. Dev Psychopathol.

[17] Kien C, Sommer I, Faustmann A, Gibson L, Schneider M, Krczal E (2019). Prevalence of mental disorders in young refugees and asylum seekers in European Countries: a systematic review. Eur Child Adolesc Psychiatry.

[18] El Baba R, Colucci E (2018). Post-traumatic stress disorders, depression, and anxiety in unaccompanied refugee minors exposed to war-related trauma: a systematic review. Int J Cult Ment Health.

[19] Reavell J, Fazil Q (2017). The epidemiology of PTSD and depression in refugee minors who have resettled in developed countries. J Ment Health.

[20] Bronstein I, Montgomery P (2011). Psychological distress in refugee children: a systematic review. Clin Child Fam Psychol Rev.

[21] Salari R, Malekian C, Linck L, Kristiansson R, Sarkadi A (2017). Screening for PTSD symptoms in unaccompanied refugee minors: a test of the CRIES-8 questionnaire in routine care. Scand J Public Health.

[22] Salari R, Malekian C, Linck L, Sarkadi A. Using CRIES-8 to screen for post-traumatic stress disorder in unaccompanied refugee minors. Eur J Public Health. 2016;26(suppl_1). Available from: 10.1093/eurpub/ckw164.007.

[23] Jensen TK, Skårdalsmo EMB, Fjermestad KW. Development of mental health problems - a follow-up study of unaccompanied refugee minors. Child Adolesc Psychiatry Ment Health. 20147;8(1):29. 10.1186/1753-2000-8-29 PMC4361195 25780387

[24] Jensen TK, Fjermestad KW, Granly L, Wilhelmsen NH (2015). Stressful life experiences and mental health problems among unaccompanied asylum-seeking children. Clin Child Psychol Psychiatry.

[25] Huemer J, Karnik N, Voelkl-Kernstock S, Granditsch E, Plattner B, Friedrich M (2011). Psychopathology in African unaccompanied refugee minors in Austria. Child Psychiatry Hum Dev.

[26] Sanchez-Cao E, Kramer T, Hodes M (2013). Psychological distress and mental health service contact of unaccompanied asylum-seeking children. Child Care Health Dev.

[27] Vossoughi N, Jackson Y, Gusler S, Stone K (2018). Mental health outcomes for youth living in refugee camps: A review. Trauma Violence Abuse.

[28] Mohwinkel L-M, Nowak AC, Kasper A, Razum O (2018). Gender differences in the mental health of unaccompanied refugee minors in Europe: a systematic review. BMJ Open.

[29] De Schrijver L, Vander Beken T, Krahé B, Keygnaert I. Prevalence of sexual violence in migrants, applicants for international protection, and refugees in Europe: A critical interpretive synthesis of the evidence. Int J Environ Res Public Health. 2018;15(9). Available from: 10.3390/ijerph15091979. 10.3390/ijerph15091979 PMC6165364 30208610

[30] Keygnaert I, Vettenburg N, Temmerman M (2012). Hidden violence is silent rape: sexual and gender-based violence in refugees, asylum seekers and undocumented migrants in Belgium and the Netherlands. Cult Health Sex.

[31] Derluyn I, Broekaert E, Schuyten G (2008). Emotional and behavioural problems in migrant adolescents in Belgium. Eur Child Adolesc Psychiatry.

[32] Hebebrand J, Anagnostopoulos D, Eliez S, Linse H, Pejovic-Milovancevic M, Klasen H (2016). A first assessment of the needs of young refugees arriving in Europe: what mental health professionals need to know. Eur Child Adolesc Psychiatry.

[33] Eurostat Press Office 2016. Almost 90 000 unaccompanied minors among asylum seekers registered in the EU in 2015 [press release].

[34] Vaughan R (2017). Oversampling in health surveys: Why, when, and how?. Am J Public Health.

[35] Sommer I, Kien C, Faustmann A, Gibson L, Schneider M, Krczal E, et al. Prevalence of mental disorders in young refugees and asylum-seekers in European countries. Eur J Public Health. 2018;28(suppl_4). Available from: 10.1093/eurpub/cky212.455. 10.1007/s00787-018-1215-z PMC6785579 30151800

[36] Perrin S, Meiser-Stedman R, Smith P (2005). The Children’s Revised Impact of Event Scale (CRIES): Validity as a screening instrument for PTSD. Behav Cogn Psychother.

[37] Royal KD (2019). Survey research methods: A guide for creating post-stratification weights to correct for sample bias. Educ Health Prof.

[38] Simmons JP, Nelson LD, Simonsohn U (2011). False-positive psychology: undisclosed flexibility in data collection and analysis allows presenting anything as significant. Psychol Sci.

[39] McLaughlin KA, Koenen KC, Hill ED, Petukhova M, Sampson NA, Zaslavsky AM (2013). Trauma exposure and posttraumatic stress disorder in a national sample of adolescents. J Am Acad Child Adolesc Psychiatry.

[40] Lewis SJ, Arseneault L, Caspi A, Fisher HL, Matthews T, Moffitt TE (2019). The epidemiology of trauma and post-traumatic stress disorder in a representative cohort of young people in England and Wales. Lancet Psychiatry.

[41] Alisic E, Zalta AK, van Wesel F, Larsen SE, Hafstad GS, Hassanpour K (2014). Rates of post-traumatic stress disorder in trauma-exposed children and adolescents: meta-analysis. Br J Psychiatry.

[42] Müller LRF, Büter KP, Rosner R, Unterhitzenberger J (2019). Mental health and associated stress factors in accompanied and unaccompanied refugee minors resettled in Germany: a cross-sectional study. Child Adolesc Psychiatry Ment Health.

[43] Jakobsen M, Meyer DeMott MA, Wentzel-Larsen T, Heir T (2017). The impact of the asylum process on mental health: a longitudinal study of unaccompanied refugee minors in Norway. BMJ Open.

[44] Thabet AA, Abu Tawahina A, El Sarraj E, Vostanis P (2008). Exposure to war trauma and PTSD among parents and children in the Gaza strip. Eur Child Adolesc Psychiatry.

[45] Eruyar S, Maltby J, Vostanis P (2018). Mental health problems of Syrian refugee children: the role of parental factors. Eur Child Adolesc Psychiatry.

[46] Thabet AA, Ibraheem AN, Shivram R, Winter EA, Vostanis P (2009). Parenting support and PTSD in children of a war zone. Int J Soc Psychiatry.

[47] Marten S. Fliehen nach Europa. Frankfurter Allgemeine Zeitung; 2019 [Internet]. Available from: https://www.faz.net/aktuell/politik/ausland/migration-flucht-der-fluechtlinge-nach-europa-16018868.html.

[48] Bean T, Derluyn I, Eurelings-Bontekoe E, Broekaert E, Spinhoven P (2007). Comparing psychological distress, traumatic stress reactions, and experiences of unaccompanied refugee minors with experiences of adolescents accompanied by parents. J Nerv Ment Dis.

[49] Vervliet M, Lammertyn J, Broekaert E, Derluyn I (2014). Longitudinal follow-up of the mental health of unaccompanied refugee minors. Eur Child Adolesc Psychiatry.

[50] Cheung KL, Ten Klooster PM, Smit C, de Vries H, Pieterse ME (2017). The impact of non-response bias due to sampling in public health studies: A comparison of voluntary versus mandatory recruitment in a Dutch national survey on adolescent health. BMC Public Health.

[51] Rothman KJ, Gallacher JEJ, Hatch EE (2013). Why representativeness should be avoided. Int J Epidemiol.

[52] Richiardi L, Pizzi C, Pearce N (2013). Commentary: Representativeness is usually not necessary and often should be avoided. Int J Epidemiol.

[53] Eurostat, the statistical office of the European Union 2019. Almost 20 000 unaccompanied minors among asylum seekers registered in the EU in 2018.

